# Features and protective efficacy of human mAbs targeting *Mycobacterium* *tuberculosis* arabinomannan

**DOI:** 10.1172/jci.insight.167960

**Published:** 2023-10-23

**Authors:** Yanyan Liu, Tingting Chen, Yongqi Zhu, Aisha Furey, Todd L. Lowary, John Chan, Stylianos Bournazos, Jeffrey V. Ravetch, Jacqueline M. Achkar

**Affiliations:** 1 Department of Microbiology and Immunology and; 2 Department of Medicine, Albert Einstein College of Medicine, Bronx, New York, USA.; 3 Department of Chemistry, University of Alberta, Edmonton, Alberta, Canada.; 4 Institute of Biological Chemistry, Academia Sinica, Taipei, Taiwan.; 5 Institute of Biochemical Sciences, National Taiwan University, Taipei, Taiwan.; 6 Public Health Research Institute at the International Center for Public Health, New Jersey Medical School – Rutgers, The State University of New Jersey, Newark, New Jersey, USA.; 7 The Rockefeller University, New York, New York, USA.

**Keywords:** Immunology, Infectious disease, Antigen, Immunoglobulins, Tuberculosis

## Abstract

A better understanding of the epitopes most relevant for antibody-mediated protection against tuberculosis (TB) remains a major knowledge gap. We have shown that human polyclonal IgG against the *Mycobacterium tuberculosis* (*M*. *tuberculosis*) surface glycan arabinomannan (AM) and related lipoarabinomannan (LAM) is protective against TB. To investigate the impact of AM epitope recognition and Fcγ receptor (FcγR) binding on antibody functions against *M*. *tuberculosis*, we isolated a high-affinity human monoclonal antibody (mAb; P1AM25) against AM and showed its binding to oligosaccharide (OS) motifs we previously found to be associated with in vitro functions of human polyclonal anti-AM IgG. Human IgG1 P1AM25, but not 2 other high-affinity human IgG1 anti-AM mAbs reactive with different AM OS motifs, enhanced *M*. *tuberculosis* phagocytosis by macrophages and reduced intracellular growth in an FcγR-dependent manner. P1AM25 in murine IgG2a, but neither murine IgG1 nor a non–FcγR-binding IgG, given intraperitoneally prior to and after aerosolized *M*. *tuberculosis* infection, was protective in C57BL/6 mice. Moreover, we demonstrated the protective efficacy of human IgG1 P1AM25 in passive transfer with *M*. *tuberculosis*–infected FcγR-humanized mice. These data enhance our knowledge of the important interplay between both antibody epitope specificity and Fc effector functions in the defense against *M*. *tuberculosis* and could inform development of vaccines against TB.

## Graphical abstract

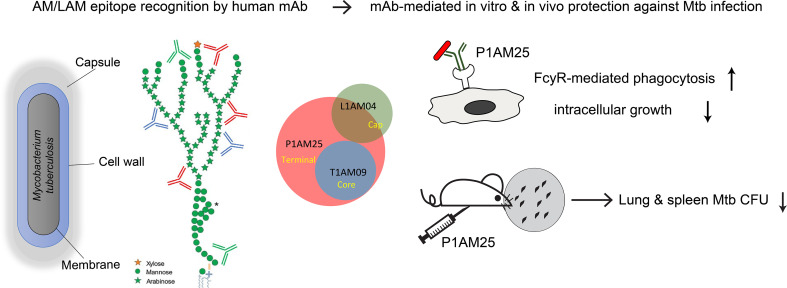

## Introduction

Active tuberculosis (TB) persists as one of the leading causes of death worldwide (1). In contrast to latent or cleared infection, the disease TB is caused by uncontrolled infection with *Mycobacterium tuberculosis* (*M*. *tuberculosis*), a predominantly intracellular pathogen with many mechanisms to escape the host’s immune response (reviewed in refs. 2, 3). In 2020, fueled by the COVID-19 pandemic, TB-associated mortality increased for the first time in decades by around 100,000 to 1.5 million deaths globally (4). In 2021, mortality increased by an additional 100,000 to now 1.6 million TB-associated deaths (1), underscoring the urgency for a more effective vaccine against TB. Efforts to develop vaccines against TB focus on inducing cell-mediated immunity but, despite recent improvements, have thus far failed to lead to a highly effective vaccine (5). Antibodies (Abs) have shown protective efficacy against *M*. *tuberculosis*, suggesting that inducing both cell-mediated and humoral immunity could be complementary (reviewed in refs. 6–12). However, a better understanding of protective antigens, their epitopes, and mechanisms of Ab-mediated protection remains a critical gap of knowledge in the TB field.

Studies suggest that Abs against surface antigens of *M*. *tuberculosis* contribute to the protection against TB (reviewed in refs. 6–12). While most surface antigens do not appear to be relevant (13), Abs targeting the surface adhesion proteins 85B and PstS1 and surface glycan arabinomannan (AM) were shown to have protective efficacy in vivo (14–21). Moreover, recent studies suggest that functions against intracellular *M*. *tuberculosis* are Fcγ receptor (FcγR) mediated (14, 15, 22). The focus of our studies is AM, a highly immunogenic glycan of the *M*. *tuberculosis* capsule and a component of the mycobacterial cell wall and membrane lipoarabinomannan (LAM) (23–25). AM and LAM play important roles in host-microbe interaction and pathogenesis of *M*. *tuberculosis* infection (reviewed in ref. 26). We and others have shown associations between human serum Abs against AM/LAM following *M*. *bovis* bacilli Calmette-Guérin (BCG) vaccination and functions against BCG (21, 27), and immunization with AM/LAM-conjugate vaccines reduced mycobacterial organ burden and/or prolonged survival of *M*. *tuberculosis*–infected mice (16–18). Passive transfer of some, but not all, murine monoclonal Abs (mAbs) against AM/LAM have shown protection against TB (19, 20, 28). However, the Ab features influencing their mechanisms and protective efficacy in vitro and in vivo are poorly understood (reviewed in ref. 29).

AM and LAM are composed of a mannan (Man) core, a highly branched arabinan (Ara) domain, and various ManLAM/AM capping motifs that can differ among mycobacterial strains (reviewed in refs. 24, 26, 29). These motifs bind to different host cell receptors (30), but whether reactivity with certain glycan motifs impacts the functions of anti-AM/LAM mAbs against *M*. *tuberculosis* is unknown. We have shown in vitro and in vivo protective efficacy of serum anti-AM IgG from asymptomatic *M*. *tuberculosis*–exposed or latently infected individuals but have not seen that efficacy with polyclonal anti-AM IgG from patients with TB (14). We further showed that polyclonal IgG reactivities to AM oligosaccharide (OS) motifs in humans with and without BCG vaccination and/or *M*. *tuberculosis* infection are tremendously heterogeneous (14, 21). Reactivity to certain capped ManAM/LAM motifs, including those with the *M*. *tuberculosis*–unique Man cap 5-deoxy-5-methylthio-xylofuranose (MTX) (31, 32), and a terminal Ara motif was associated with in vitro IgG functions with sera from asymptomatic BCG-vaccinated individuals and individuals with presumed latent *M*. *tuberculosis* infection (LTBI). However, establishing causation of the antigenic determinants within AM that are most relevant in eliciting protective Abs requires study with structurally defined mAbs.

Based on our findings with polyclonal IgG, we hypothesized that Ab reactivity to certain AM OS structures, specifically terminal Ara motifs and those with Man capping, influences the functions of human mAbs against *M*. *tuberculosis*. We previously reported 2 human mAbs with high affinity to AM, T1AM09 (aka AM009) and L1AM04, which we isolated via single–B cell sorting to capsular *M*. *tuberculosis* AM with peripheral blood mononuclear cells (PBMCs) from 2 asymptomatic individuals with *M*. *tuberculosis* exposure or presumed LTBI (33). These 2 mAbs recognized different glycan epitopes, distinct from other anti-AM/LAM mAbs reported (reviewed in ref. 29). Using single–B cell sorting to AM and PBMCs of an additional asymptomatic individual with a positive tuberculin skin test (TST) and interferon-γ release assay (IGRA), and a reported history of childhood TB, we report here the isolation and characterization of a human mAb named P1AM25. This mAb has a very high affinity to AM and reacts with a diverse set of AM OS motifs, including Man capped motifs with and without MTX and terminal Ara motifs. With the 3 high-affinity human mAbs recognizing different AM OS motifs, our main objective was to investigate the impact of AM epitope specificity and FcγR binding on the functions of anti-AM IgG mAbs against *M*. *tuberculosis*. Our secondary objective was to assess FcγR-humanized (FcγR-hu) mice as a model for TB. We show that in vitro and in vivo IgG mAb functions are influenced by both reactivity to AM OS motifs and FcγR-mediated effects. In addition to demonstrating protective effects with passive transfer of mouse IgG2a P1AM25 in the classical murine TB model, we demonstrate that FcγR-hu mice can serve as a TB model ideally suited for testing human Ab efficacy against TB. Importantly, we show protective in vivo effects of human IgG1 P1AM25 against *M*. *tuberculosis* in this humanized murine model. These data enhance our knowledge of the importance of AM epitope specificity in combination with FcγR-mediated effects for Ab functions against *M*. *tuberculosis* and could inform development strategies for vaccines and immunotherapies against TB.

## Results

### A high-affinity human mAb binds to multiple and distinct AM OS motifs.

**Figure 1:**
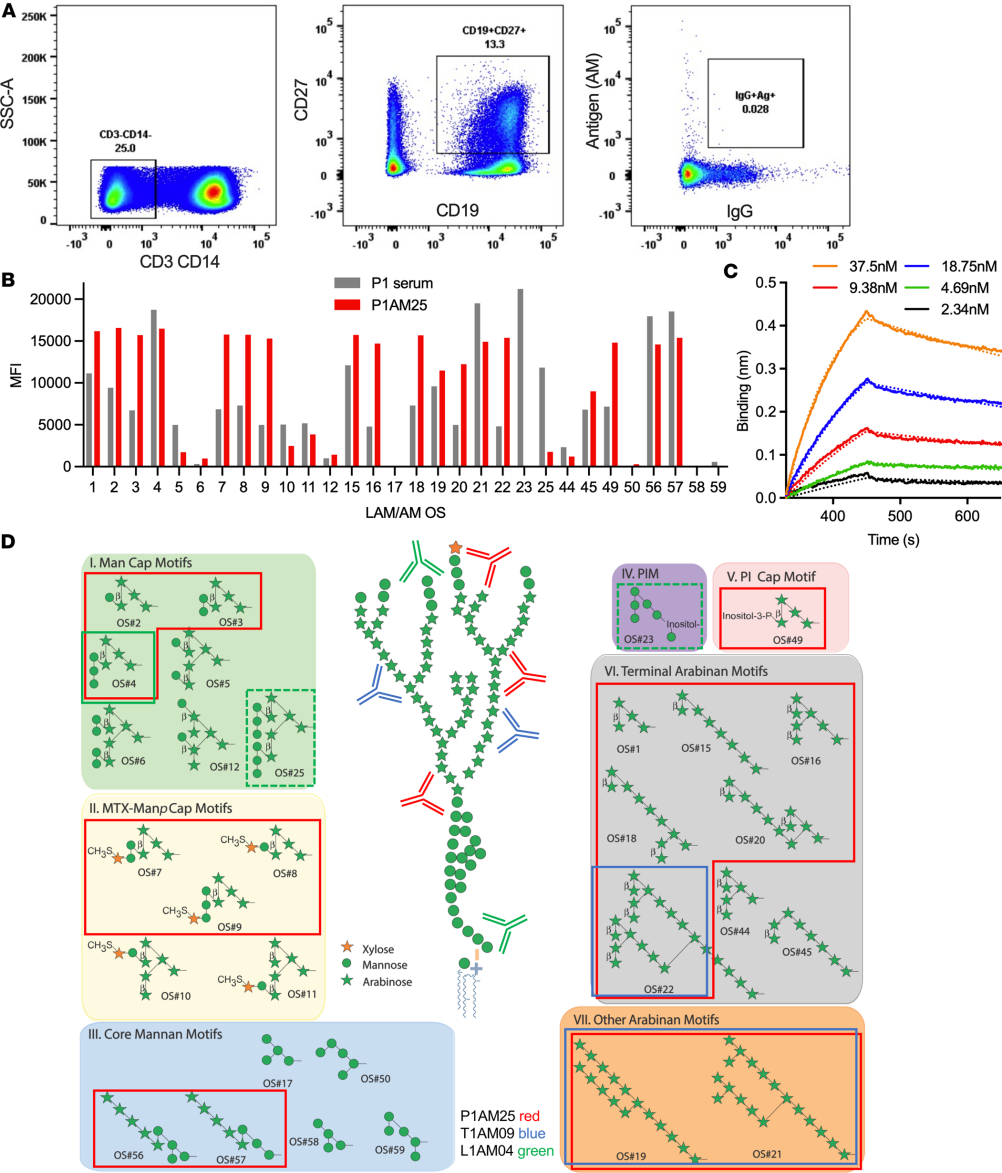
The human mAb P1AM25 reacts strongly with diverse and distinct glycan motifs within AM/LAM. (**A**) Gating of IgG^+^AM^+^ B cells from PBMCs of participant P1 by fluorescence-activated cell sorting (full set of gating images shown in Supplemental Figure 1). (**B**) Polyclonal serum IgG reactivity of participant P1 (1:100; gray bars) and mAb P1AM25 (1 μg/mL; red bars) to 30 synthetic mycobacterial AM OS motifs. Median fluorescence intensity (MFI) is shown for AM OS with numbers corresponding to position on glycan array. Specifically, P1AM25 reacts strongly with the following OS motifs organized by groups according to location within AM as shown in **C** and framed in red (adapted from refs. 29, 52): I) mannose-capped (Man Cap) motifs Man_1_Ara_4_ (OS#2), Man_2_Ara_4_ (OS#3), and Man_3_Ara_4_ (OS#4); II) MTX-Man*p* cap motifs MTX_1_Man_2_Ara_4_ (OS#7), MTX_1_Man_1_Ara_4_ (OS#8), and MTX_1_Man_3_Ara_4_ (OS#9); III) core Man motifs Ara_5_Man_4_ (OS#56) and Ara_5_Man_3_ (OS#57); V) myoinositol-phosphate (PI) cap motif PI_1_Ara_4_ (OS#49); VI) terminal Ara motifs Ara_4_ (OS#1), Ara_8_ (OS#15), Ara_7_ (OS#16), Ara_10_ (OS#18), Ara_11_ (OS#20), and Ara_22_ (OS#22); and VII) Core Ara Motifs Ara_16_ (OS#19) and Ara_18_ (OS#21). (**C**) Binding curve of P1AM25 to capsular AM (isolated from H37Rv) by BLI. *K_D_* = 3.4 × 10^–9^ M. Experimental data are shown as solid lines, and statistically fitted curves are shown as dashed lines. (**D**) Structural OS motifs organized into groups based on location within AM/LAM (adapted from refs. 29, 30, 72). OS motifs strongly recognized by P1AM25 are framed in red and for comparison shown for the human anti-AM mAbs T1AM09 (blue) and L1AM04 (green). Solid lines represent strong and dashed weak binding (glycan array of T1AM09 and L1AM04 performed in ref. 33). Data are representative of 2 independent experiments.

**Figure 2:**
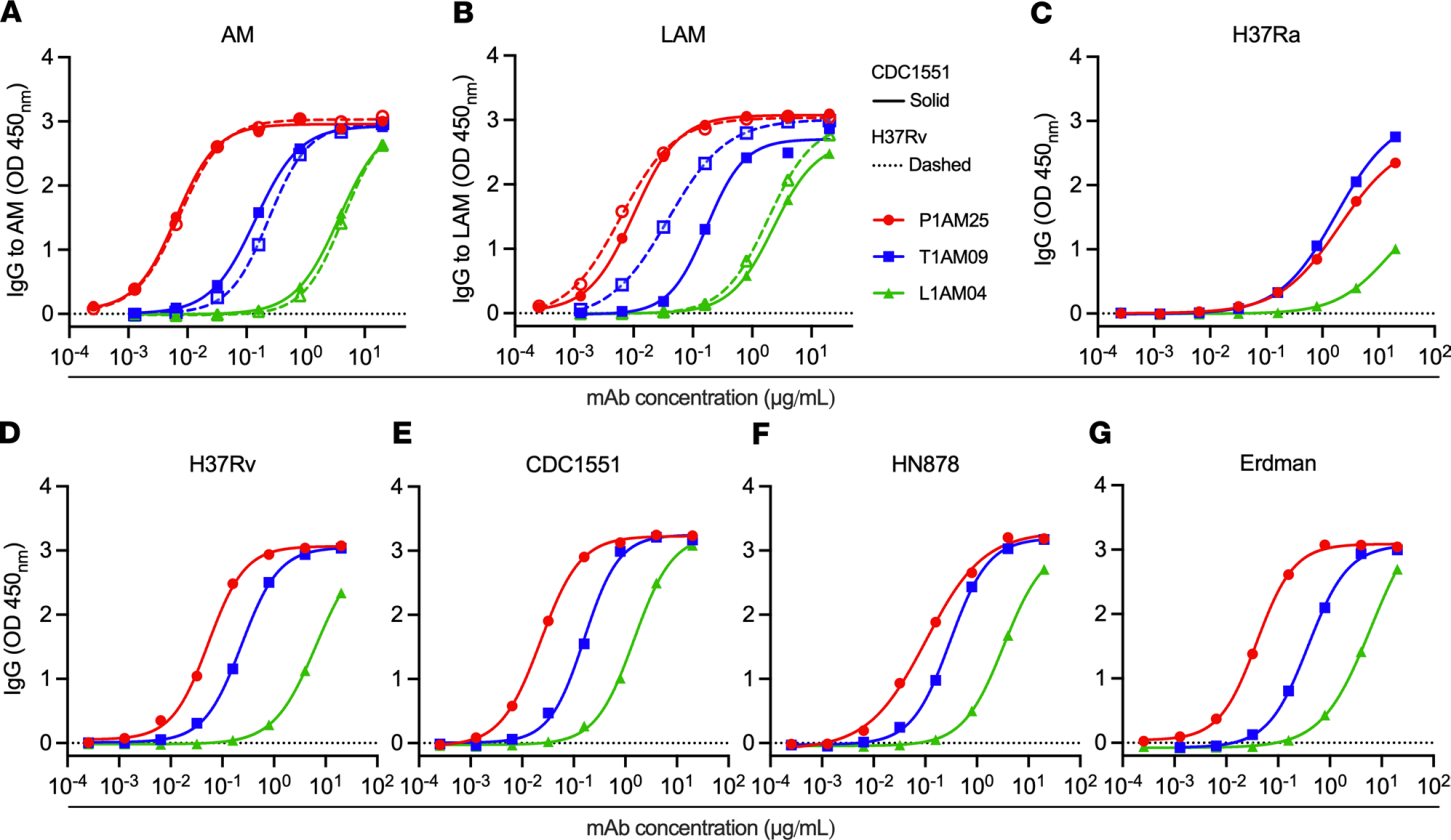
Human mAb P1AM25 shows strong binding to AM/LAM and *M*. *tuberculosis* strains. (**A**) Binding of P1AM25 (red) in comparison with T1AM09 (blue) and L1AM04 (green) to *M*. *tuberculosis* capsular AM and (**B**) LAM of H37Rv and CDC1551 determined by ELISA (AM and LAM coated at 10 μg/mL). Binding of anti-AM mAbs to whole bacteria of (**C**) avirulent H37Ra and virulent (**D**) H37Rv and (**G**) Erdman *M*. *tuberculosis* lab strains, and clinical *M*. *tuberculosis* strains (**E**) CDC1551 and (**F**) HN878 (Beijing), assessed by ELISA. Bacteria were grown stationary and without detergent to preserve the capsule. All ELISA data are representative of 2 independent experiments.

To broaden our repertoire of anti-AM mAbs with reactivity to distinct glycan epitopes for the study of Ab features influencing protective functions against *M*. *tuberculosis*, we performed single–B cell sorting to *M*. *tuberculosis* AM with PBMCs from participant P1 (Figure 1A and Supplemental Figure 1; supplemental material available online with this article; https://doi.org/10.1172/jci.insight.167960DS1). P1 was selected due to persistently high serum IgG reactivity to AM at different time points with recognition of a wide range of AM OS motifs (Figure 1B). P1 was an asymptomatic adult who had immigrated to the United States from India several years before, reported a possible history of childhood TB meningitis (which was treated), and had a positive TST and IGRA (QuantiFERON-TB Gold In-Tube) at the time of blood collection. Of expressed mAbs from 12 IgG- and AM-positive memory B cells, P1AM25 was found to have very high affinity to *M*. *tuberculosis* capsular AM by bio-layer interferometry (BLI) with an equilibrium dissociation constant (*K_D_*) value in the low nanomolar range (*K_D_* = [3.4 ± 0.06] × 10^–9^ M, association constant [*k_on_*] = [3.5 ± 0.04] × 10^5^/ms, dissociation constant [*k_off_*] = [1.2 ± 0.01] × 10^–3^/s; Figure 1C). This affinity was higher than that for most anti-glycan mAbs reported (34, 35), including those of our previously isolated anti-AM mAbs T1AM09 (*K_D_* = [2.6 ± 0.04] × 10^–8^ M) and L1AM04 (*K_D_* = [9.6 ± 0.56] × 10^–8^ M) (33). Binding specificity, determined by a glycan array containing 30 synthetically generated AM OS motifs (30), showed that P1AM25 recognized a range of AM OS motifs, different from T1AM09 and L1AM04 and distinct from other anti-AM/LAM mAbs (Figure 1D) (29, 33). Specifically, P1AM25 reacted strongly with several Man capped motifs, including those containing the *M*. *tuberculosis* (in this position) distinctive MTX residue (31, 32, 36). P1AM25 further reacted with several terminal and a few core Ara motifs. Moreover, P1AM25 is the first anti-AM/LAM mAb reported to react with the core mannan motifs Ara_5_Man_4_ (OS#56) and Ara_5_Man_3_ (OS#57) (29). Reactivity of P1AM25 to structures of other major mycobacterial surface glycans, including α-glucan, trehalose mycolates and lipooligosaccharides (LOSs), phenolic glycolipids (PGLs), phosphatidyl-myoinositol mannosides (PIMs), and glycopeptidolipids (GPLs), on the glycan array was not detected (30) (Supplemental Figure 2). Compared with human anti-AM mAbs T1AM09 and L1AM04, and consistent with the higher affinity by BLI (Figure 1C) (33), P1AM25 also showed stronger binding to capsular AM and cell wall LAM purified from different *M*. *tuberculosis* strains as well as whole *M*. *tuberculosis* bacteria from the most geographically widespread lineages 2 (Beijing HN878) and 4 (H37Rv, CDC1551, Erdman) (37), followed by T1AM09 and then L1AM04 (Figure 2).

### Enhanced M. tuberculosis phagocytosis by anti-AM mAbs is influenced by reactivity to certain AM motifs.

**Figure 3:**
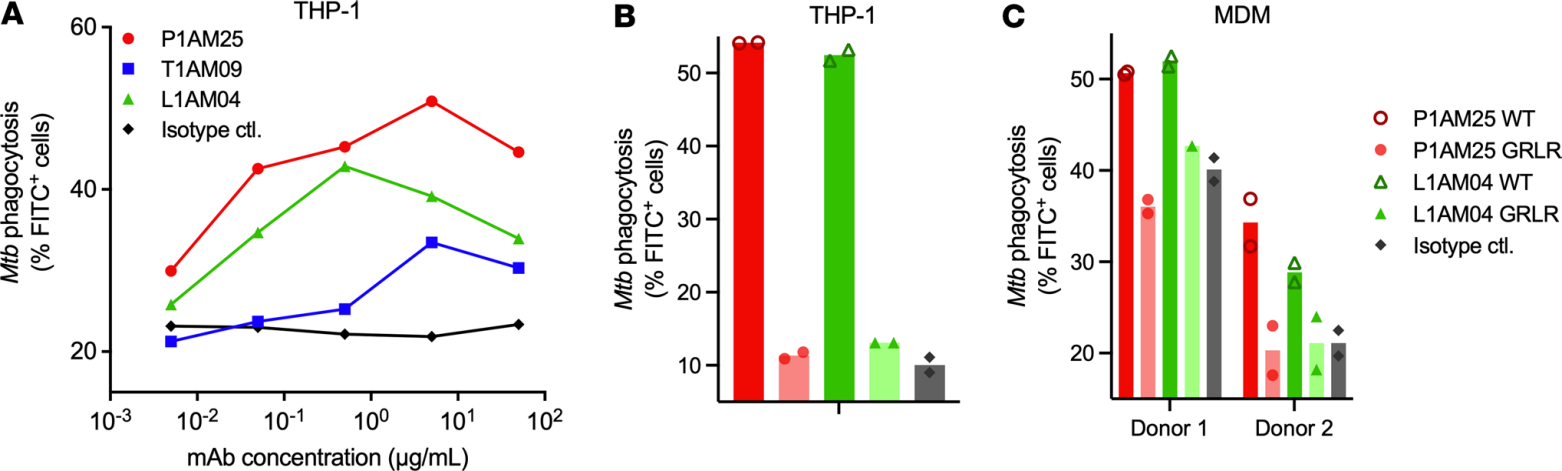
Enhanced macrophage *M*. *tuberculosis* phagocytosis by anti-AM mAbs is influenced by both reactivity with AM motifs and FcγR binding. (**A**) *M*. *tuberculosis* (H37Ra; multiplicity of infection [MOI] 20) phagocytosis by THP-1–derived human macrophages in the presence of human IgG1 P1AM25 (red), T1AM09 (blue), and L1AM04 (green) at various mAb concentrations. Dots represent means of duplicates. Data are representative of 2 independent experiments. Effect of P1AM25 and L1AM04 (5 μg/mL) in human IgG1 and human IgG1 non–FcγR-binding GRLR mutant on *M*. *tuberculosis* (H37Ra) phagocytosis by THP-1–derived human macrophages (**B**, MOI 20) or human MDMs (**C**, MOI 10) from 2 healthy donors. Isotype control mAb is human IgG1 (gray). Columns represent mean of duplicates (circles/diamonds/triangles).

To investigate whether, beyond affinity, reactivity to specific AM OS motifs influences mAb functions against *M*. *tuberculosis*, we first examined the impact of our 3 anti-AM mAbs, expressed in the same human IgG1 vector in human embryonic kidney (HEK) cells, on *M*. *tuberculosis* phagocytosis. Despite the lower binding of mAbs to H37Ra compared with H37Rv and other virulent *M*. *tuberculosis* strains (Figure 2), we performed these experiments with the avirulent *M*. *tuberculosis* strain H37Ra for practical reasons because it allowed us to assess phagocytosis at various mAb concentrations under biosafety level 2 (BSL2) conditions. Both P1AM25 and L1AM04 enhanced *M*. *tuberculosis* (H37Ra) phagocytosis by THP-1 macrophages in a concentration-dependent manner (Figure 3A). The effect diminished as Ab concentrations went above 10 μg/mL, presumably due to a prozone-like effect. We saw enhanced phagocytosis starting at mAb concentrations as low as 0.1 μg/mL, indicating that using H37Ra, rather than virulent *M*. *tuberculosis* strains, had little impact on our results. Although T1AM09 had higher affinity to AM/LAM and avidity to whole bacteria than L1AM04, it had little effect on *M*. *tuberculosis* phagocytosis. L1AM04, on the other hand, despite its lower affinity to AM and lower avidity to various *M*. *tuberculosis* strains, particularly H37Ra, showed a similar effect on *M*. *tuberculosis* uptake as P1AM25, a result that was verified with human monocyte-derived macrophages (MDMs; Figure 3), suggesting that AM epitope specificity influences mAb-mediated *M*. *tuberculosis* phagocytosis.

### Enhanced M. tuberculosis phagocytosis by anti-AM mAbs is FcγR mediated.

To assess the extent by which the enhanced *M*. *tuberculosis* phagocytosis is influenced by Fc-FcγR interactions, we investigated P1AM25 and L1AM04 in human IgG1 (wild-type) and the human IgG1 non–FcγR-binding GRLR mutant (38) (Figure 3, B and C). Both mAbs lost their effect when expressed in the GRLR vector and coincubated with *M*. *tuberculosis* and THP-1 or human MDMs, showing that the enhanced *M*. *tuberculosis* phagocytosis was FcγR mediated.

### P1AM25 inhibits M. tuberculosis intracellular growth and enhances phagosome-lysosome fusion.

**Figure 4:**
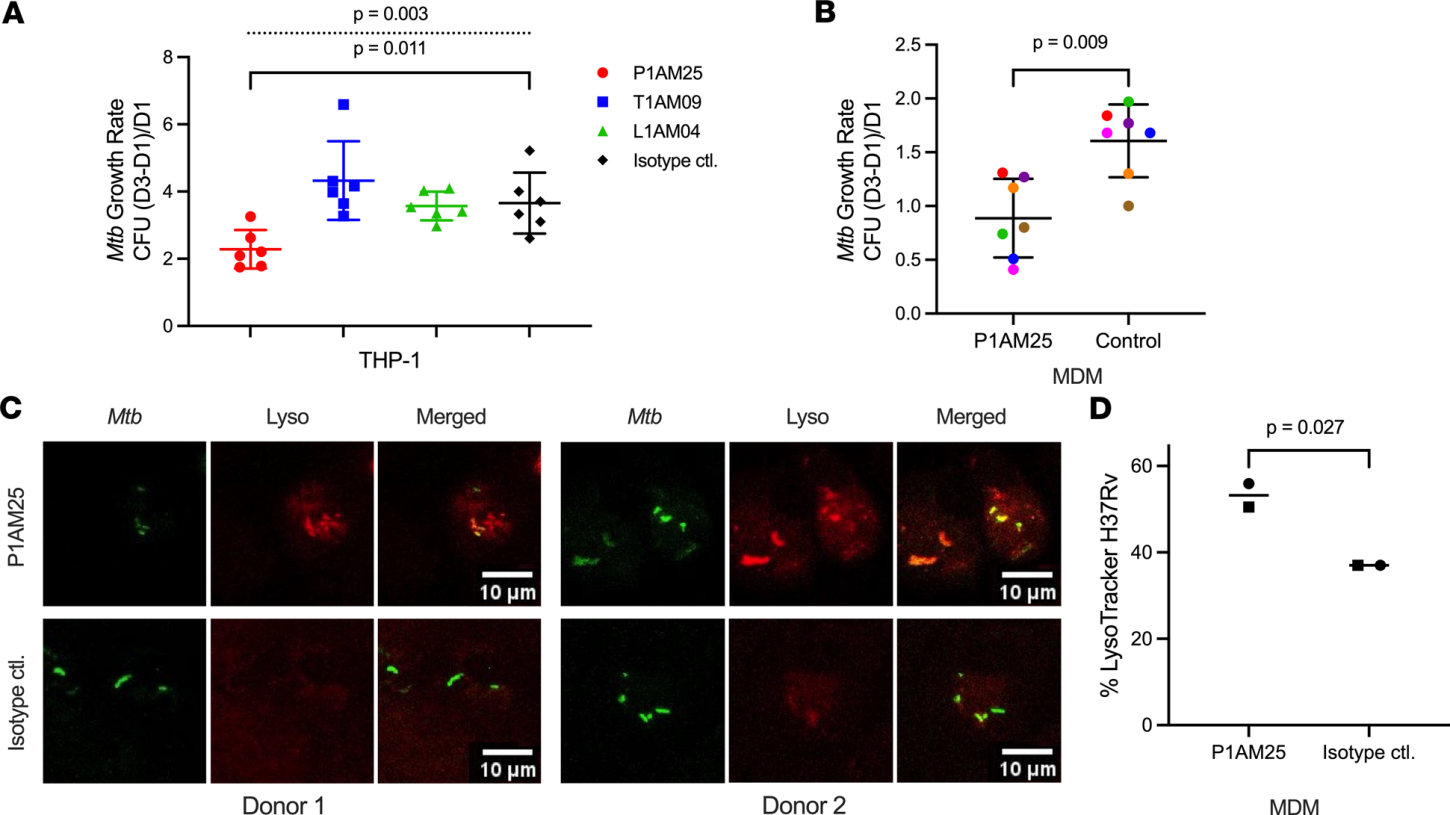
*M*. *tuberculosis* intracellular growth is reduced in the presence of mAb P1AM25 but not T1AM09 or L1AM04 and is mediated by enhanced P-L fusion in human macrophages. (**A**) *M*. *tuberculosis* (H37Rv; MOI 1) intracellular growth rates (CFU of [Day3 – Day1]/Day1) with anti-AM human IgG1 mAbs (5 μg/mL) in THP-1–differentiated human macrophages. An irrelevant human IgG1 mAb is included as an isotype control. Dots and error bars represent mean ± SD of sextuplicates. Data are representative of 2 independent experiments. Dashed line represents 1-way ANOVA. Solid line represents 2-tailed *t* test. (**B**) *M*. *tuberculosis* (H37Rv; MOI 1) intracellular growth rates in the presence of P1AM25 or control mAb (5 μg/mL) in blood MDMs from 7 different healthy donors. Dots are means of triplicate wells. Error bars represent mean ± SD of 7 donors. Individual colors shown for each donor with P1AM25 correspond to data for the same donor with isotype control mAb. Paired 2-tailed *t* test. (**C**) Confocal microscopy images of human MDMs from 2 healthy donors incubated with P1AM25 or an isotype-matched irrelevant IgG1 control mAb (5 μg/mL), followed by infection with Alexa Fluor 488–conjugated H37Rv and labeled with LysoTracker probes. Images show *M*. *tuberculosis* (green) localized within phagosomes, lysosomes (red), and colocalization of *M*. *tuberculosis*/phagosomes and lysosomes (yellow) indicating P-L fusion. (**D**) Degree of P-L fusion enhancement by P1AM25 compared with irrelevant isotype-matched control mAb (5 μg/mL). Circles and squares each represent mean P-L fusion data (from triplicate or duplicate wells) from the MDMs of the 2 healthy donors. *T* test. P-L, phagosome-lysosome.

Increased phagocytosis is not necessarily to the benefit of the host because *M*. *tuberculosis* grows well within macrophages by preventing phagosome maturation (39). We therefore investigated the effects of our mAbs on intracellular *M*. *tuberculosis* growth, which, to account for differences in mAb-mediated phagocytosis, was assessed as growth rate. P1AM25, but neither T1AM09 nor L1AM04, reduced *M*. *tuberculosis* intracellular growth in THP-1–derived human macrophages when compared with a human IgG1 irrelevant control mAb (*P* < 0.05; Figure 4A). We verified the *M*. *tuberculosis* growth-reducing effect of P1AM25 with human MDMs from 7 healthy donors (*P* < 0.01; Figure 4B). To further investigate one of the potential mechanisms by which P1AM25 could reduce *M*. *tuberculosis* intracellular growth, we examined the extent of P-L fusion in *M*. *tuberculosis*–infected MDMs. With MDMs from 2 of the donors for whom we had sufficient cells left, we showed that P1AM25 compared with a control mAb enhanced P-L fusion by 36%–51% (*P* < 0.05; Figure 4, C and D).

### P1AM25 shows protective in vivo efficacy in a traditional murine TB model.

**Figure 5:**
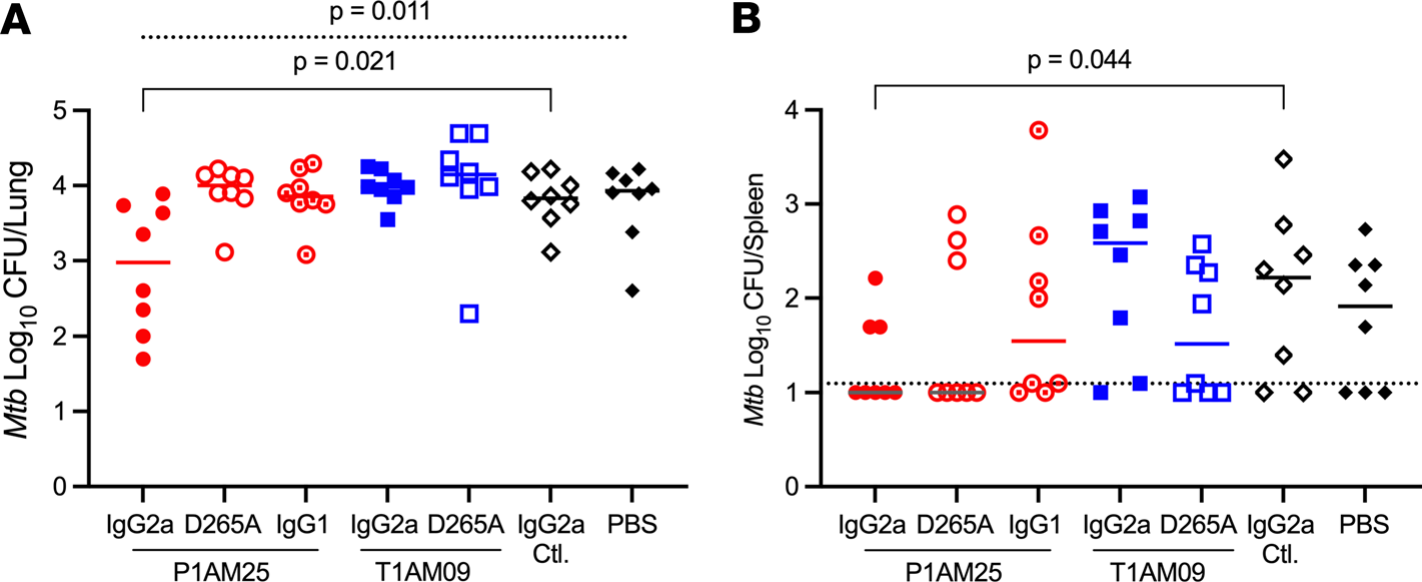
P1AM25 in murine IgG2a but neither IgG1 nor non–FcγR-binding variant IgG1D265A nor any T1AM09 isotype shows protective efficacy in *M*. *tuberculosis*–infected C57BL/6 mice. Effects of passive transfer of P1AM25 and T1AM09 murine isotypes in low-dose *M*. *tuberculosis*–infected (Erdman; mean lung CFU 51 ± 15 one day postinfection) C57BL/6 mice. MAbs (50 μg per mouse) were given i.p. 1 day prior to and 7 days after aerosol infection. Lines represent median (**A**) lung and (**B**) spleen CFU 2 weeks after infection. Dashed line shows the level of detection limit (12.5 CFU). Kruskal-Wallis group comparison test for lung CFU (dashed line), followed by targeted 2-group comparison with Mann-Whitney *U* test (solid line). To account for several mice with spleen CFU below the limit of detection, a Tobit model was used.

To determine the protective effect of anti-AM mAbs in vivo, we performed passive transfer experiments with C57BL/6 mice, a strain widely used as a TB model (40). To evaluate both the relevance of antigen-binding and Fc effector functions, we first expressed the 2 highest affinity mAbs P1AM25 and T1AM09 in murine IgG2a and the non–FcγR-binding murine IgG1D265A mutant (41). To compare mouse IgG isotypes that differ in inducing effector functions, we further expressed P1AM25 in murine IgG1. Of note, through different FcγR affinities, murine IgG1 mediates more inhibitory while IgG2a strongly promotes activating effector functions (41, 42) (Supplemental Table 1). MAbs were given intraperitoneally (i.p.) 1 day before infection (50 μg) and, to account for the half-lives of murine mAbs (43), 7 days postinfection (50 μg) to mice infected with low-dose aerosolized *M*. *tuberculosis* (~50 colony-forming units [CFU]). We used H37Rv for most of our in vitro experiments because it is one of the most widely used strains of *M*. *tuberculosis* and allowed comparison to our prior in vitro data with polyclonal IgG (14). However, we used Erdman for the aerosol infection of mice because it grows faster than H37Rv in many in vivo models and is more virulent (44, 45), allowing us to see more pronounced differences in mAb passive transfer experiments at early *M*. *tuberculosis* infection time points. Compared with an irrelevant IgG2a control, P1AM25 in murine IgG2a but neither D265A nor IgG1 showed a significant reduction in lung bacterial burden 2 weeks after infection (*P* = 0.02; Figure 5A). Regarding T1AM09, neither IgG2a nor the D265A mutant showed protection. Although not all mice had detectable spleen CFU by 2 weeks, more mice in the P1AM25 murine IgG2a had an undetectable CFU compared with the irrelevant control IgG2a mAb (5/8 versus 2/8; *P* = 0.04; Figure 5B). Interestingly, there were also 5/8 mice in the D265A mutant group that had undetectable spleen CFU, raising the hypothesis that P1AM25 could reduce *M*. *tuberculosis* dissemination irrespective of FcγR binding.

### FcγR-hu mice can serve as a valuable TB model for the investigation of human Abs.

**Figure 6:**
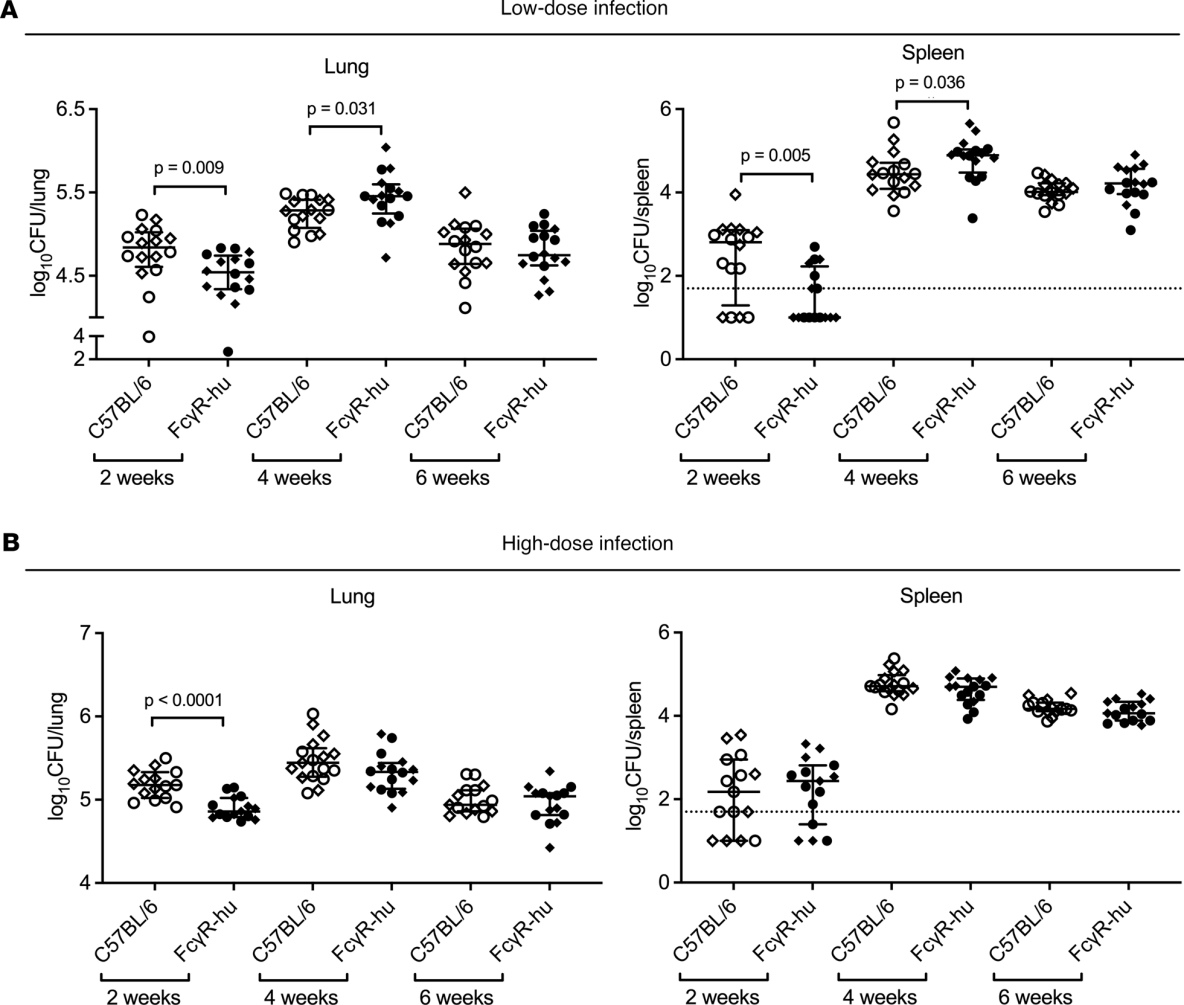
FcγR-hu mice have comparable bacterial burden to C57BL/6 mice at 4 and 6 weeks after *M*. *tuberculosis* infection. CFU of lung and spleen after (**A**) low-dose *M*. *tuberculosis* (Erdman) infection (mean lung CFU 78 ± 13 one day postinfection) or (**B**) high-dose *M*. *tuberculosis* infection (mean lung CFU 300 ± 39 one day postinfection). Lines and error bars represent medians with interquartile ranges (IQRs). Diamonds represent female mice; circles represent male mice. Mann-Whitney *U* test.

Although the FcγR family is largely conserved between mice and humans, there are substantial variations in FcγR structure and expression patterns that restrict the analysis of human Ab functions and effects in the murine model (reviewed in refs. 46, 47). We therefore assessed the course of *M*. *tuberculosis* infection in a recently generated FcγR-hu mouse strain (48). In these mice with C57BL/6 background, all endogenous mouse FcγR genes have been deleted and replaced with the entire human FcγR family. To assess our mAbs in human IgG isotypes with an in vivo model that more closely resembles human immunology, we first investigated whether FcγR-hu mice can serve as a TB model comparable to that of the commonly used C57BL/6 wild-type mice (40). After aerosolized infection with low- (~80 CFU) or high-dose (~300 CFU) *M*. *tuberculosis* (Erdman), the organ bacterial burden increased from 2 to 4 weeks and declined from 4 to 6 weeks in both C57BL/6 and FcγR-hu mice, comparable to other *M*. *tuberculosis* infection studies in C57BL/6 mice (Figure 6). At 2 weeks postinfection, in both the low- and high-dose infection, we observed lower lung CFU in the FcγR-hu compared with the C57BL/6 wild-type mice (*P* < 0.01). This difference was also observed in the spleen at 2 weeks postinfection in the low-dose but not the high-dose infection. By contrast, at 4 weeks in the low-dose infection experiment, FcγR-hu mice had slightly but significantly higher lung and spleen CFU (*P* < 0.05), whereas no differences were seen in the high-dose infection experiment. Of note, this small difference in lung CFU between C57BL/6 mice and FcγR-hu mice was not significant in a repeated low-dose infection experiment (~70 CFU) at 4 weeks. At 6 weeks, no organ CFU differences were seen between the strains. Moreover, no significant differences in organ bacterial burden were observed between male and female mice in either of the strains in the initial 4 weeks following *M*. *tuberculosis* infection (Supplemental Figure 3).

In both WT and FcγR-hu mice we saw low IgG and IgM responses to *M*. *tuberculosis* membrane fractions and even lower responses to *M*. *tuberculosis* lysates (data not shown) 2–4 weeks after aerosolized *M*. *tuberculosis* infection with only a moderate increase at week 6, which was significant in C57BL/6 mice (Supplemental Figure 4). Histological studies at 2 weeks postinfection revealed that both the *M*. *tuberculosis–*infected FcγR-hu and WT mice displayed alveolar infiltration of immune cells consisting of macrophages, neutrophils, and lymphocytes as part of the lung granulomatous response. While these infiltrates occupied less than 1% of the area of the lung sections examined for both strains of mice, the cellular aggregates appeared more robust in the WT mice (Supplemental Figure 5, A and B). Of note, the granulomatous reaction of the FcγR-hu mice also exhibited perivascular and peribronchiolar lymphocytic infiltrates that were devoid of macrophages or neutrophils. The pale staining regions of these lymphocytic aggregates were reminiscent of germinal center features (Supplemental Figure 5, C and E), which represent areas occupied by proliferating B cells. Such germinal center–like aggregates were absent in the lungs of tuberculous WT animals (Supplemental Figure 5, D and F). Interestingly, as the infection progressed, the germinal center–like aggregates in the lungs of the *M*. *tuberculosis–*infected FcγR-hu mice receded, being no longer apparent at 4 and 6 weeks postinfection, and were not seen at 4 and 6 weeks in the WT mice (data not shown). No major differences were observed between male and female mice.

To assess the timeline for the generation of murine anti-human Abs, we evaluated sera from male and female C57BL/6 and FcγR-hu *M*. *tuberculosis*–infected mice (150 CFU *M*. *tuberculosis* Erdman) after weekly i.p. injections of 200 μg human commercially obtained IgG. Compared with the PBS control group, we observed a 2-fold titer increase in murine anti-human IgG and IgM titers in all groups after 4–5 weeks of human IgG injections (Supplemental Figures 6 and 7). Collectively, despite lower bacterial burden at week 2, these data show that FcγR-hu mice share similar *M*. *tuberculosis* infection course with C57BL/6 mice and can serve as a human model for the investigation of mAbs against *M*. *tuberculosis*.

### Protective effect of P1AM25 in FcγR-hu mice.

**Figure 7:**
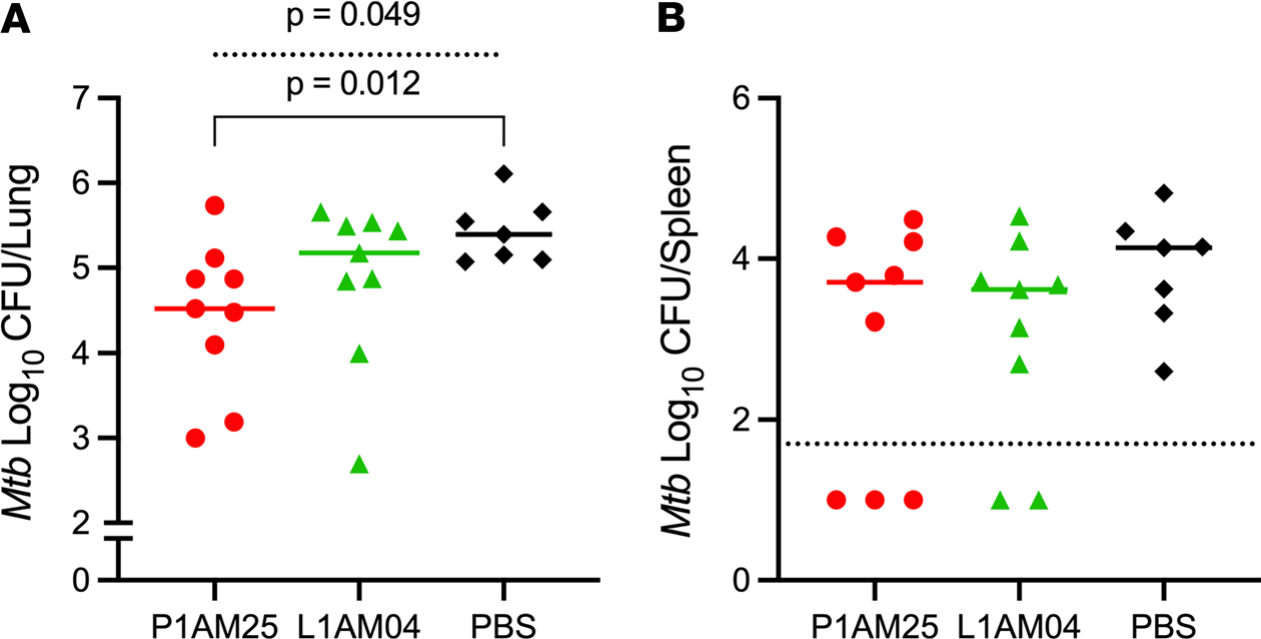
Human IgG1 P1AM25 shows protective efficacy in *M*. *tuberculosis*–infected FcγR-hu mice. Effect of P1AM25 and L1AM04 human IgG1 in low-dose *M*. *tuberculosis*–infected (Erdman; mean lung CFU 18 ± 4.6 one day postinfection) FcγR-hu mice. MAbs (50 μg per mouse) were given i.p. 1 day prior to and 10 days after aerosol infection. Lines represent median (**A**) lung and (**B**) spleen CFU 3 weeks after infection. Dashed line in **B** shows the level of detection limit for spleen (50 CFU). Data are representative of 2 independent experiments. Dashed line in **A** represents Kruskal-Wallis test. Solid line in **A** represents Mann-Whitney *U* test.

Given the similar *M*. *tuberculosis* infection course in FcγR-hu and C57BL/6 wild-type mice and the protection of murine P1AM25 IgG2a against TB in C57BL/6 mice, we investigated the protective efficacy of human P1AM25 IgG1 in *M*. *tuberculosis*–infected FcγR-hu mice. Because T1AM09 showed neither in vitro nor in vivo protective effects against *M*. *tuberculosis* in prior experiments, we included only L1AM04 in human IgG1 for comparison. Because of the lower *M*. *tuberculosis* burden in FcγR-hu compared with C57BL/6 mice at 2 weeks postinfection (Figure 6), we assessed mAb efficacy at 3 weeks after low-dose *M*. *tuberculosis* infection. Consistent with the data in C57BL/6 wild-type mice, human P1AM25 IgG1 reduced *M*. *tuberculosis* lung burden by around 1 log compared with control (*P* < 0.05; Figure 7A). Although mice receiving human L1AM04 IgG1 had slightly lower lung CFU than control mice, this difference was not statistically significant. All the control mice had a detectable spleen CFU. On the other hand, although not statistically significant, 3/9 in the P1AM25 and 2/9 mice in the L1AM04 group had undetectable spleen bacterial burden (Figure 7B). Together with the in vitro data and results with wild-type C57BL/6 mice, our data with FcγR-hu mice demonstrate the protective efficacy of the human anti-AM/LAM mAb P1AM25 against *M*. *tuberculosis*.

## Discussion

Motifs within bacterial surface glycans serve as key targets for Ab-inducing glycoconjugate vaccines against several deadly human pathogens (49, 50), but detailed knowledge about their role in *M*. *tuberculosis* infection is lacking. We have recently shown that polyclonal IgG from LTBI individuals against the *M*. *tuberculosis* surface glycan AM has protective in vitro and in vivo efficacy against *M*. *tuberculosis* and that reactivity to specific AM/LAM OS motifs was associated with in vitro functions (14). To understand the causal relationship between AM epitope recognition and Ab functions against *M*. *tuberculosis*, we here isolated a high-affinity human anti-AM mAb that recognizes various AM motifs, including all OS motifs that were previously found to correlate with functions of polyclonal anti-AM IgG. Investigating this mAb, P1AM25, in human IgG1 together with 2 other human anti-AM mAbs that exhibit different AM OS binding profiles (33), we show that both Ab reactivity with specific AM motifs and FcγR binding is critical for Ab-mediated protection against TB.

The various key roles of AM and LAM in *M*. *tuberculosis* infection include the promotion of *M*. *tuberculosis* uptake and its survival in macrophages (reviewed in ref. 26). These are facilitated by interacting with various host cell receptors, such as the mannose receptor (MR), and the inhibition of phagosomal maturation. Although the roles of specific AM/LAM motifs in *M*. *tuberculosis* infection remain incompletely understood, many of the host cell receptors interact primarily with mannose or Man capped motifs (30). In our previous work we found serum IgG reactivity to capped ManLAM motifs to be associated with enhanced *M*. *tuberculosis* and BCG phagocytosis by human macrophages (14, 21). Specifically, for individuals with LTBI, these were Man_1-3_Ara_4_, linked with the *M*. *tuberculosis* distinctive MTX residue (31, 32, 36, 51), and included Man_2_Ara_4_ without MTX, which overlapped between individuals with LTBI and, in a different study, with recent BCG vaccination. For individuals with LTBI, reactivity to the terminal Ara_4_ motif without Man/MTX capping was also found. While our human mAb P1AM25 recognized all these motifs as well as additional terminal Ara motifs, our human mAb L1AM04 recognized only the capped structure Man_3_Ara_4_. The enhanced *M*. *tuberculosis* phagocytosis by human macrophages in the presence of human IgG1 P1AM25 and L1AM04 but not T1AM09 suggests that reactivity with capped ManLAM/AM and/or terminal Ara motifs is more important for this Ab-mediated function than reactivity with other Ara motifs. However, the intracellular *M*. *tuberculosis* growth reduction as well as protective in vivo efficacy in *M*. *tuberculosis*–infected mice in the presence of P1AM25 but neither L1AM04 nor T1AM09 indicates that reactivity with Man_3_Ara_4_, or just 1 capped motif, may be insufficient for protective functions against *M*. *tuberculosis*. Interestingly, recently one of the few other human anti-LAM IgG1 mAbs reported, isolated from a patient with TB and reactive with Ara_4/6_ with/without Man_1_ with/without MTX (29, 52), did not impact *M*. *tuberculosis* intracellular growth in human macrophages (28). While further studies with mAbs reacting with more defined and distinct AM epitopes will allow more granular investigations, these data suggest that reactivity with several specific capped and/or terminal/core Ara motifs promotes protective efficacy of anti-AM/LAM IgG. Given the successful development of semi- and fully synthetic carbohydrate vaccines that target specific glycan motifs and are now in preclinical and clinical studies (50), these data could have implications for TB vaccine development strategies.

Engagement of different FcγRs can alter the course of *M*. *tuberculosis* infection in mice (53). Moreover, in recent studies, certain IgG Fc glycosylation and FcγR-mediated effects were associated with functions of polyclonal human LTBI IgG against the predominantly intracellularly located *M*. *tuberculosis* (13, 14, 22). However, aside from our work with anti-AM–specific polyclonal IgG (14), other studies investigated total serum IgG and IgG reactive with multiple mycobacterial antigen preparations, such as purified protein derivative, thus limiting conclusions of Fc relevance with antigen-specific IgG (13, 22). A recent study with mAbs against the mycobacterial protein PstS1 showed Fc-dependent mAb effects against *M*. *tuberculosis* in an ex vivo human whole blood assay (15). However, passive transfer studies demonstrating the FcγR-mediated effects in vivo and studies with human mAbs against other *M*. *tuberculosis* antigens, especially those against AM epitopes, are lacking. We demonstrate here that in addition to reactivity with certain AM epitopes, FcγR-mediated effects are critical for in vitro and in vivo efficacy of anti-AM mAbs. These data suggest that P1AM25 can redirect the uptake of *M*. *tuberculosis* by mannose and other host cell receptors to uptake of the *M*. *tuberculosis*-mAb complex by activating FcγRs. We show that at very low doses (50 μg per mouse) of murine IgG2a, but neither a non–FcγR-binding mutant nor the murine IgG1, P1AM25 has protective efficacy in the classical C57BL/6 TB model.

We further demonstrate the protective efficacy of human IgG1 P1AM25 in *M*. *tuberculosis*–infected FcγR-hu mice and show that these mice can serve as a model for the investigation of human IgG against TB. Interestingly, we observed a lower lung CFU in FcγR-hu mice compared with WT C57BL/6 mice at 2 weeks after *M*. *tuberculosis* infection but did not see these differences at 4 and 6 weeks postinfection. The underlying pathophysiology is unclear to us. FcγR-hu mice mount similar murine Ab responses to immunogens compared to WT B6 mice (48), and we showed that this was also true for Abs against *M*. *tuberculosis*. Because, as anticipated, anti–*M*. *tuberculosis* Abs were low in both FcγR-hu and WT mice within the first weeks after *M*. *tuberculosis* infection, we believe that the difference seen at 2 weeks is unlikely due to FcγR signaling involving *M*. *tuberculosis*–specific Abs. Histological studies, however, showed that 2 weeks after infection with *M*. *tuberculosis*, the FcγR-hu mice appeared to have less lung inflammation and exhibited the feature of germinal center formation, a difference that became less noticeable at 4 and 6 weeks. Although the mechanisms that result in the discrepant granulomatous response in the 2 mouse strains are currently unclear, the data suggest that early after *M*. *tuberculosis* infection, signaling via human and murine FcγR in the mouse may result in distinct development of the granulomatous response that resolves later in the course of infection.

One of the well-known strategies of *M*. *tuberculosis* to escape the host cell surveillance is by limiting the fusion of bacteria containing intracellular phagosomes with lysosomes (54). Early studies have shown that coating *M*. *tuberculosis* with BCG-vaccinated rabbit serum prior to infection of murine macrophages can subvert the inhibition of P-L fusion caused by *M*. *tuberculosis* (55). A later study showed that both *M*. *tuberculosis* LAM and AM but not lipomannan or mannan were responsible for the P-L fusion restriction (56). This restriction could be reversed by blocking the MR on monocytes and macrophages and by coincubating LAM-coated beads with the anti-LAM murine mAb CS35, which is reactive with a range of AM/LAM OS motifs (52, 56). Consistent with these data and our anti-AM–specific polyclonal IgG data (14), we show that the protective mechanisms of P1AM25 included enhancing intracellular P-L fusion. The *M*. *tuberculosis* growth reduction in the presence of P1AM25 but neither T1AM09 nor L1AM04 adds further insights and suggests that reactivity to several ManLAM caps and/or terminal Ara motifs is needed to block the restriction of P-L fusion mediated by *M*. *tuberculosis* AM and LAM. However, investigating molecular pathways leading to the P-L fusion restriction was beyond the scope of the current studies. Detailed studies, ideally with even more mAbs reacting with additional defined and diverse AM epitopes, are needed to investigate the role of AM/LAM motifs in the survival of *M*. *tuberculosis* within macrophages.

While we here provide evidence that a human mAb against AM/LAM protects against *M*. *tuberculosis* infection, our study was limited to 3 different anti-AM mAbs, restricting a detailed analysis of the various potential roles and mechanisms the blocking of specific AM/LAM motifs could have. We have further focused on showing binding to lab and clinical *M*. *tuberculosis* strains from 2 modern and geographically the most widespread lineages to date (Beijing HN878 from lineage 2, and Erdman, H37Rv and CDC1551, from lineage 4) (37). Investigating potentially diverse binding of our mAbs to all *M*. *tuberculosis* lineages, ideally with a broader panel of anti-AM mAbs with even more distinct binding profiles, is warranted for future studies. Moreover, the small number of mAbs limited associations between mAb kinetics and functions. Nevertheless, we show that the high-affinity anti-AM mAb T1AM09, which binds to whole-cell *M*. *tuberculosis* and is reactive with Ara motifs different from capped ManLAM or terminal Ara motifs (33), had fewer functions than the mAb L1AM04, which is of lower affinity but is reactive with a specific ManLAM cap motif (Man_3_Ara_4_). These data support that binding to specific AM motifs influences mAb functions, irrespective of the binding kinetics.

In conclusion, we show here that a high-affinity human mAb against the mycobacterial surface glycan AM, P1AM25, is reactive with epitopes previously associated with functions of human polyclonal IgG and is protective against TB in an FcγR-dependent manner. We further show that FcγR-hu mice are an invaluable model for investigating the role of human IgG against TB. These data enhance our knowledge of antigen and epitope specificity critical for Ab-mediated immunity against TB and could inform TB vaccine and immunotherapy development strategies. Additional studies with a broad spectrum of mAbs reacting with more defined and distinct AM epitopes are now needed to dissect the detailed roles specific motifs and their blocking have in *M*. *tuberculosis* infection.

## Methods

### Human samples

Serum and PBMCs of participant P1, used for single–B cell sorting, were collected under a TB study (#2006-428), and leukopaks, obtained for assays with macrophages from anonymous human donors, were obtained from the New York Blood Center. PBMCs were isolated by density gradient separation using Ficoll-Paque and frozen in liquid nitrogen until use (57).

### M. tuberculosis strains

*M. tuberculosis* strains H37Ra, H37Rv, Erdman, CDC1551, and Beijing (HN878) were grown in Middlebrook 7H9 broth supplemented with 10% (vol/vol) oleic albumin dextrose catalase enrichment and 0.5% (vol/vol) glycerol, with or without 0.05% tyloxapol. For AM generation, bacteria were then subcultured into minimal media comprising 1 g/L KH_2_PO_4_, 2.5 g/L Na_2_HPO_4_, 0.5 g/L asparagine, 50 mg/L ferric ammonium citrate, 0.5 g/L MgSO_4_ × 7 H_2_O, 0.5 mg/L CaCl_2_, 0.1 mg/L ZnSO_4_, and 0.1% (vol/vol) glycerol, pH 7.0 (16, 58).

### Mycobacterial antigens

Capsular AM was isolated and purified from the *M*. *tuberculosis* strains as described (16, 25, 59). In brief, bacteria were incubated at 37°C for 21 days in horizontally placed roller bottles containing minimal media without any detergents to preserve the formation of the capsule. The capsule was then collected by physically disrupting bacterial cells using glass beads (VWR). The protein portion was removed from the capsular extract, and AM was separated from other glycans by column chromatography. LAM purified from H37Rv was obtained from the Biodefense and Emerging Infectious Research Resources Repository (BEI Resources; NR-14848). LAM purified from CDC1551 was obtained from Delphi Chatterjee at Colorado State University (Fort Collins, Colorado, USA) (60).

### Single–B cell sorting and isolation of mAbs against AM

AM-specific human B cells were isolated from PBMCs by standard B cell immunophenotyping and fluorescence-activated cell sorting followed by single-cell B cell receptor cloning (33, 61, 62). Briefly, purified *M*. *tuberculosis* capsular AM was first activated by 1-cyano-4-dimethylaminopyridinium tetrafluoroborate and then conjugated to biotin with EZ-Link Amine-PEG3-Biotin (Thermo Fisher Scientific) (63). PBMCs were probed by biotinylated AM together with the Ab cocktail, followed by a second staining with phycoerythrin-labeled streptavidin (405204, BioLegend) to identify the AM-positive cells. Zombie Aqua (423101, BioLegend) was included to exclude dead cells. Single live B cells that were AM^+^CD3^–^CD14^–^CD19^+^CD27^+^IgG^+^ (317306, 367116, 302216, 356417, 410711, BioLegend) were sorted by a FACSAria high-speed cell sorter (Becton Dickinson) into a 96-well plate containing catch buffer. Plates were stored at –80°C until cloning.

Complementary DNA was synthesized from lysed single B cells by reverse transcription PCR. Ig heavy and light chain variable region (V-region) genes were amplified using a 2-step nested PCR, then sequenced (GENEWIZ, Azenta Life Sciences). The PCR products were further amplified by cloning PCR, followed by the Gibson assembly that cloned Ig genes into IgG1 heavy and light chain expression vectors (61, 62). The vectors were cotransfected and expressed in HEK293 cells to produce mAbs (61, 62). Ab V-region genes were additionally cloned into murine IgG2a and IgG1D265A, human GRLR expression vectors, and expressed in expi293T cells (Thermo Fisher Scientific) as previously described (38) for in vitro and in vivo assays. Germline alleles were determined using the IMGT database (http://imgt.org).

### MAb reactivity to glycan motifs

The reactivity of mAbs to AM epitopes was determined by a glycan microarray comprising 61 synthetically generated OS fragments from 7 mycobacterial surface glycans: AM/LAM, α-glucan, trehalose mycolates and LOSs, PGLs, PIMs, and GPLs (30, 33). Briefly, slides were blocked with blocking buffer at 4°C overnight, followed by incubation with mAbs at 1 or 5 μg/mL. MAb binding to OS was detected by biotinylated goat anti-human IgG (109-065-088, Jackson ImmunoResearch Laboratories) and then probed by a streptavidin-SureLightP3 Cy5 (16695, Cayman Chemical). The GenePix 4000 Microarray scanner system (Molecular Devices) was used for scanning, and images were analyzed by GenePix Pro 7. The reactivity of mAbs to glycan OS fragments was measured as MFI, which consisted of subtracting the background pixel intensity from the median pixel intensity of each spot.

### Ab binding assays

MAb binding to AM assay was performed by BLI using the OctectRed system (ForteBio, Pall) (33). Briefly, purified capsular AM was biotinylated and immobilized onto streptavidin-coated sensors. The sensor was then dipped into wells containing 1 mAb at serial dilutions to create binding curves and calculate the *K_D_*. The binding of AM mAbs to purified capsular AM and intact bacterial surface was further quantified by ELISA (33). Briefly, purified AM was coated on 96-well microtiter plates (Maxisorp, Thermo Fisher Scientific) at 10 μg/mL in 50 μL. For whole-cell ELISA, *M*. *tuberculosis* strains grown in media without detergent were coated on 96-well plates overnight and blocked with BLOTTO (Thermo Fisher Scientific) (33). Serially diluted mAbs were then added to the antigen- or bacteria-coated wells, and the bound mAb was probed with horseradish peroxidase–conjugated goat anti-human IgG Fc (2048-05, SouthernBiotech), followed by TMB-ELISA chromogenic substrate (Thermo Fisher Scientific). The reaction was stopped by adding an equal volume of 2N sulfuric acid (MilliporeSigma), and the optical densities (ODs) were measured at 450 nm subtracted by 540 nm. A human IgG1 mAb against a non–*M*. *tuberculosis* antigen was included as a negative control.

### Functional mAb assays

#### Phagocytosis assay.

Ab-mediated effects on *M*. *tuberculosis* phagocytosis were assessed with human macrophages (14, 21). Briefly, human THP-1 monocytic leukemia cells (ATCC) were differentiated into macrophages by phorbol 12-myristate 13-acetate and infected with FITC-labeled *M*. *tuberculosis* strain H37Ra at an MOI of 20 for 3 hours in the presence of mAbs and 10% heat-inactivated (HI) fetal bovine serum (FBS). Cells were then washed, detached, and treated with trypan blue to quench the fluorescence from noninternalized, membrane-bound, FITC-labeled bacteria. The rate of *M*. *tuberculosis* phagocytosis was determined by flow cytometry. A human IgG1 mAb against a non–*M*. *tuberculosis* antigen was included as negative control. For functions with human MDMs, PBMCs from anonymous donors were plated into 48-well plates at 1 × 10^6^ cells per well and differentiated into MDMs after adding macrophage colony-stimulating factor at 10 ng/mL for 7 days (14, 64, 65). Cells were infected with FITC-labeled H37Ra (MOI 10), and phagocytosis rate was determined as for THP-1–derived macrophages.

#### M. tuberculosis intracellular growth inhibition assay.

Effects of mAbs on intracellular *M*. *tuberculosis* growth were determined in human macrophages (14). Briefly, THP-1 cell–derived macrophages were infected with *M*. *tuberculosis* H37Rv at an MOI of 1 for 2 hours in the presence of mAbs and 10% HI FBS. The cells were washed extensively after infection to remove any extracellular bacteria and afterward lysed (day 1) or refilled with fresh growth medium containing the same mAb and lysed after 48 hours (day 3). Quantity of bacteria was determined by plating serial dilutions on Middlebrook 7H10 agar plates. CFU were counted after a 4-week incubation at 37°C. Results were validated with human MDMs from 7 healthy individuals. Because intracellular growth can be influenced by many conditions, such as different mAbs during the initial infection leading to different degrees of *M*. *tuberculosis* uptake and outcomes, we calculated the bacterial growth rate as CFU = (day 3 – day 1)/day 1, which also allows for an easy comparison across different studies (14).

#### P-L fusion.

P-L fusion was determined as described (14). Briefly, human MDMs were infected with Alexa Fluor 488–conjugated H37Rv at an MOI of 5 in the presence of mAbs (5 μg/mL) and 10% HI FBS for 3 hours at 37°C. After washing, cells were probed with 100 nM LysoTracker Red DND-99 (Life Technologies) for 1 hour at 37°C. Slides were then fixed with 2% paraformaldehyde and sealed in mounting media (VECTASHIELD, Vector Laboratories) using Cytoseal 60 (Thermo Fisher Scientific). Images were taken using a Leica SP8 confocal microscope, and the percentage of phagosomes that colocalized with the LysoTracker was quantified by counting 100–400 bacteria-containing phagosomes by Volocity (Quorum Technologies), an image analysis software that can quantify entities and their overlap and has been used to assess P-L fusion (66, 67).

### Assessment of FcγR-hu mice as a TB model

FcγR-hu mice were previously generated (48). Briefly, C57BL/6 mice were genetically modified by sequential insertion of *loxP* sites on chromosome 1 followed by Cre recombinase–mediated deletion, resulting in the knockout of murine FcγRIIB, FcγRIII, and FcγRIV (48). The resulting mice were crossed to a strain lacking murine FcγRI on chromosome 4. The progeny were then crossed with transgenic C57BL/6 mice expressing human FcγRI, FcγRII, FcγRIIB, FcγRIIIA, and FcγRIIIB on all immune cells. All newborns were PCR genotyped at 7 loci to ensure correct mouse FcγR deletion and human FcγR expression.

To compare the classical TB model with C57BL/6 mice (Jackson Laboratory) and FcγR-hu mice, mice that were 10–15 weeks old were age and sex matched in each experiment. Using an exposure aerosol chamber custom-fitted to a class III biosafety cabinet (Baker), mice were infected by aerosol with low-dose (~70 CFU) and high-dose (~300 CFU) of *M*. *tuberculosis* strain Erdman. The low-dose *M*. *tuberculosis* infection experiment was repeated twice. Five mice were sacrificed the day after infection to establish the infection dose, and groups of 7–8 mice were sacrificed 2, 4, and 6 weeks postinfection. During necropsy, lungs and spleens were removed, homogenized in PBS, and plated on selective Middlebrook 7H11 agar with 10 mg/L amphotericin B, 50 mg/L carbenicillin, 200 U/mL polymyxin B, and 20 mg/L trimethoprim (7H11 selective agar). The plate CFU were counted after 3–4 weeks of incubation at 37°C. The left lung lobes were removed and fixed in 10% neutral buffered formalin (Thermo Fisher Scientific) for histology. Tissues were embedded with paraffin, sectioned at 5 μm thickness, and stained with hematoxylin and eosin stain. Three lung sections per mouse were analyzed. Slides were scanned with a PerkinElmer P250 High-Capacity Slide Scanner.

### Murine Ab investigations

To determine the development of anti-human Ab responses in *M*. *tuberculosis*–infected mice, total human IgG was purified from commercially obtained human sera (Gemini Bio Products) using protein G agarose according to manufacturer’s instructions (Thermo Fisher Scientific). C57BL/6 and FcγR-hu mice (8–10 weeks old) were injected i.p. with 200 μg human IgG in 200 μL PBS 1 day before and weekly after *M*. *tuberculosis* infection. Control mice received PBS i.p. The mice were infected aerogenically with approximately 150 CFU of *M*. *tuberculosis* (Erdman). Blood was collected prior to passive IgG transfer and *M*. *tuberculosis* infection (week 0), followed by weekly blood draws via saphenous vein, or, at necropsy, through cardiac puncture, and serum was filtered twice through sterile 0.22 μm Spin-X centrifuge tubes (Corning) before being taken out of the BSL3.

For mouse anti-human Ab ELISAs, pooled sera from 5 FcγR-hu and 5 C57BL/6 mice with high anti-human Abs were diluted 1:100 and run as reference samples on each plate. Any plate with reference samples’ OD 405 nm falling outside 2 SD was rerun. Murine sera (1:200) were added and serially diluted 1:2 until a final dilution of 1:102,400 was reached. Alkaline phosphate–conjugated goat anti-mouse IgG (1031-04, SouthernBiotech) and rat anti-mouse IgM (1140-04, SouthernBiotech) were used as secondary Abs. After a 1-hour incubation at 37°C, P-nitrophenyl phosphate (PNPP; Thermo Fisher Scientific) substrate was added to each plate, and after another hour, the absorbance was read at 405 nm. Assays were repeated on 2 separate days.

To establish positive cutoff OD values for the anti-human IgG and IgM titers, sera from reference mice (20 wild-type and 20 FcγR-hu mice, 10 of each sex, 8–10 weeks old) neither exposed to human IgG nor exposed to *M*. *tuberculosis* were investigated via ELISA as outlined above. For each mouse strain and isotype, the cutoff OD value was determined by using the mean + 2 SD OD at serum dilution of 1:200.

Ab endpoint titers were determined by the reciprocal of the highest dilution that gave a positive reading above the cutoff value (68). Since 1 additional serial dilution could give a substantial difference in the anti-human Ab titer, we used a curve-fitting method to determine interdilution titers (69). All dilutions were log-transformed and plotted against their respective dilution OD values on a 4-point sigmoidal curve. The anti-human IgG and IgM titers of each mouse at each time point were interpolated using the cutoff value for that respective group. If the OD value at the starting dilution of 1:200 was at or below the cutoff, the titer for that time point was recorded as 200. A 2-fold increase from the baseline titer at week 0 was considered seroconversion.

To determine the development of anti–*M*. *tuberculosis* Ab responses in *M*. *tuberculosis*–infected mice, murine serum samples diluted 1:100 were added in duplicates to the H37Rv membrane fraction-coated (MEM, BEI Resources; NR-14831) wells. Previously quantified murine anti–*M*. *tuberculosis* high- and low-titer sera (1:100) from prior in-house experiments were used as controls on individual plates. Since heat shock protein X (HspX) is found within *M*. *tuberculosis* cell membrane fractions (70, 71), CS-49, a monoclonal anti–*M*. *tuberculosis* HspX Ab (BEI Resources; NR-13814), was used to ensure consistent coating of *M*. *tuberculosis* MEM and used as an additional positive control. Alkaline phosphate–conjugated goat anti-mouse IgG and rat anti-mouse IgM were used as secondary Abs. PNPP was added to each plate, and each assay was read at 405 nm after 1 hour of incubation. Assays were repeated on 2 separate days.

### Passive transfer experiments

C57BL/6 female mice (7–8 weeks old, The Jackson Laboratory) were used to study murine mAbs, and FcγR-hu male and female mice (7–8 weeks old) (48) were used for human mAbs. Mice were infected aerogenically with a low dose of *M*. *tuberculosis* strain Erdman. Wild-type mice were injected i.p. with 50 μg of mAbs in 200 μL PBS 1 day before and 7 days after *M*. *tuberculosis* infection and FcγR-hu mice with 50 μg of mAbs 1 day before and 10 days after *M*. *tuberculosis* infection. Control mice received PBS alone or an irrelevant mouse IgG2a mAb. Necropsies were performed 2 weeks after *M*. *tuberculosis* infection of wild-type and 3 weeks after *M*. *tuberculosis* infection of FcγR-hu mice. To determine the bacterial burden, lungs and spleens were aseptically removed and homogenized in PBS. Serial dilutions of the homogenates were plated on 7H11 selective agar. CFU were counted after 3–4 weeks of incubation at 37°C.

### Statistics

GraphPad Prism version 8 and SAS version 9.4 were used to perform statistical analysis. Depending on the data distribution, we used Kruskal-Wallis test or 1-way ANOVA for multiple-group comparisons and Mann-Whitney *U* or paired or unpaired *t* test for 2-group comparisons. All statistical tests used were 2 tailed, and a *P* value less than 0.05 was considered significant. For each analysis of human mAbs reactive with different AM/LAM epitopes, we presented the *P* value from the omnibus test (Kruskal-Wallis or ANOVA) to determine whether there were any statistically significant differences in distribution or means across groups. Following a statistically significant overall test, we also presented a *P* value for the planned pairwise contrast corresponding to our primary comparison of interest (the mAb P1AM25 reactive with AM/LAM epitopes associated in our prior studies with protection against TB using polyclonal serum Abs) (14). Conventionally, as only 1 planned test was performed, family-wise error rates are not a serious concern (as they are with post hoc testing), and we did not adjust for multiple testing. We agree that an omnibus test is not strictly needed given our planned (as opposed to post hoc) contrasts; however, we opted to present both results because we included other human anti-AM/LAM mAbs reactive with different AM/LAM epitopes in our studies. To examine differences in spleen bacterial burden between experimental groups, we estimated a Tobit model to account for the CFU counts below the limit of detection in the spleens of several mice in some of the mAb groups at 2 weeks postinfection. This approach models a latent variable for true bacterial burden that is assumed to be normally distributed but left censored at the lower limit of detection.

### Study approval

#### Human ethics statement.

Blood samples of the adult P1 were collected under a TB study (#2006-428), approved by the institutional review board of the Albert Einstein College of Medicine/Montefiore Medical Center. Written informed consent was obtained prior to enrollment and blood draw.

#### Animal ethics statement.

All wild-type and FcγR-hu mouse studies were approved by the Institutional Animal Care and Use Committee at the Albert Einstein College of Medicine under protocols 00001210 and 0000136.

### Data availability

The accession numbers for the nucleotide and protein sequences for variable regions of P1AM25 are GenBank OP896195 and OP896196. Glycan array data were deposited in the NCBI Gene Expression Omnibus database with accession number GSE218873. Values for all data points in graphs are reported in the Supporting Data Values file.

## Author contributions

YL, TC, and JMA conceived the study. YL, TC, YZ, TLL, SB, JVR, and JMA developed methodology. YL, TC, YZ, AF, and JMA investigated. YL, TC, YZ, JC, SB, JVR, and JMA analyzed data. TLL, SB, JVR, and JMA provided resources. JMA acquired funding. JMA supervised. YL, TC, YZ, and JMA wrote the original manuscript. YL, TC, YZ, AF, TLL, JC, SB, JVR, and JMA reviewed and edited the manuscript. YL and TC provided equal first author contributions to the work. First author order was determined as follows: under supervision of JMA, YL in collaboration with TC designed and performed the experiments and analyzed the data; YL created the graphs and wrote most of the manuscript with input from TC and JMA.

## Supplementary Material

Supplemental data

Supporting data values

## Version 1 09/21/2023

In-Press Preview

## Version 2 10/23/2023

Electronic publication
