# Supplementary material for: Features and protective efficacy of human mAbs targeting Mycobacterium tuberculosis arabinomannan
Source: JCI Insight. 2023 Oct 23;8(20):e167960. doi: 10.1172/jci.insight.167960 (PMC10619501; doi:10.1172/jci.insight.167960)
Supplement: Supplemental data [file jciinsight-8-167960-s172.pdf]

Supplemental Materials:

Supplemental Table 1. Relative binding of different mouse IgG subclasses to FcgRs. Adapted from (41, 42).

Binding of mouse IgG subclasses to FcγR					
**Subclass**	**Activating FcγRI**	**Inhibitory FcγRIIb**	**Activating FcγRII**	**Activating FcγRIV**	**A/I Ratio**
**IgG1**	-	++	++	-	**0.1 (III/IIb)**
**IgG1 D265A**	-	-	-	-	
**IgG2a**	++++	+	+	+++	**69 (IV/IIb)**

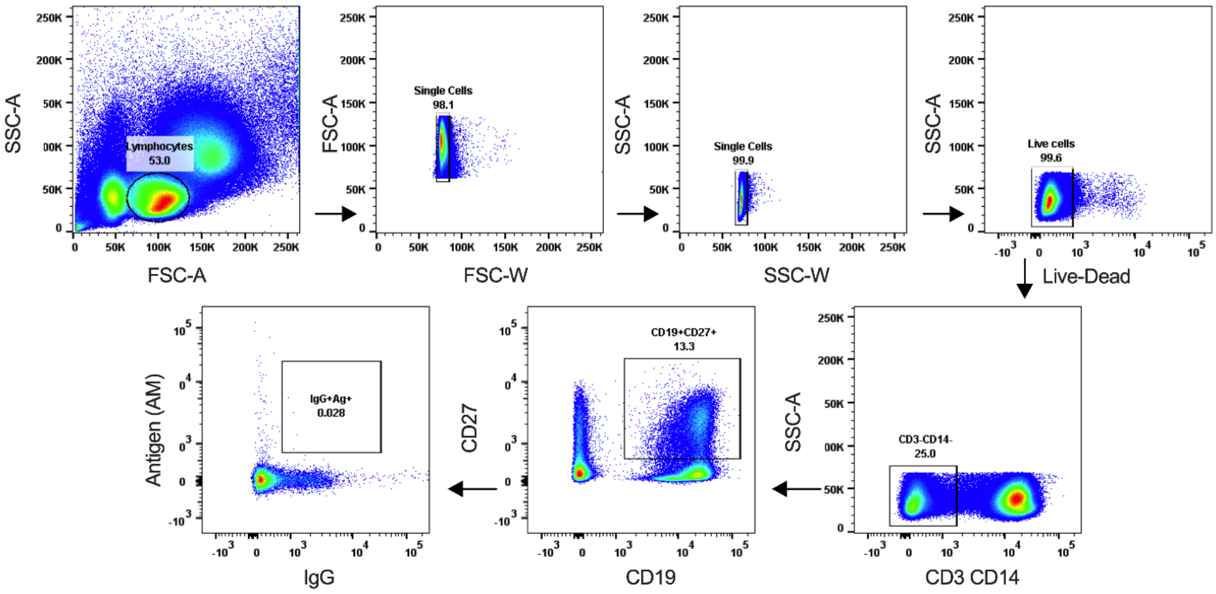

Supplemental Figure 1. Sorting strategy of AM-specific human B cells. Gating of CD3-CD14-CD19+CD27+IgG+AM+ B cells from PBMCs of subject P1 by fluorescence activated cell sorting.

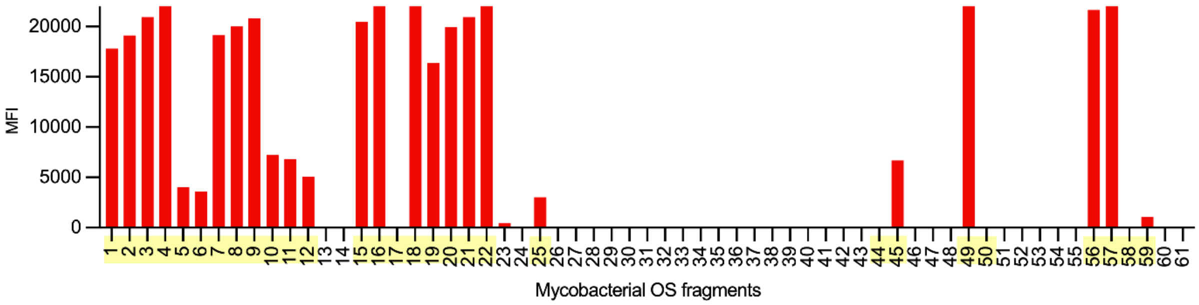

Supplemental Figure 2. P1AM25 reacts strongly to OS motifs within AM/LAM but not other major mycobacterial glycans. Median fluorescent intensity (MFI) of P1AM25 (5 µg/mL) to 61 synthetic mycobacterial oligosaccharide (OS) motifs. Reactivity is shown for OS fragments with numbers corresponding to position on glycan array ((30); α-glucan [OS#13, 14, 24, 46, 48, 52], LOS [OS#38, 39, 54, 55], PGLs [OS#26-37, 40-43, S51, 53], PIMs [OS#23] and GPLs [OS#47, 60, 61]. AM/LAM specific fragments are highlighted in yellow.

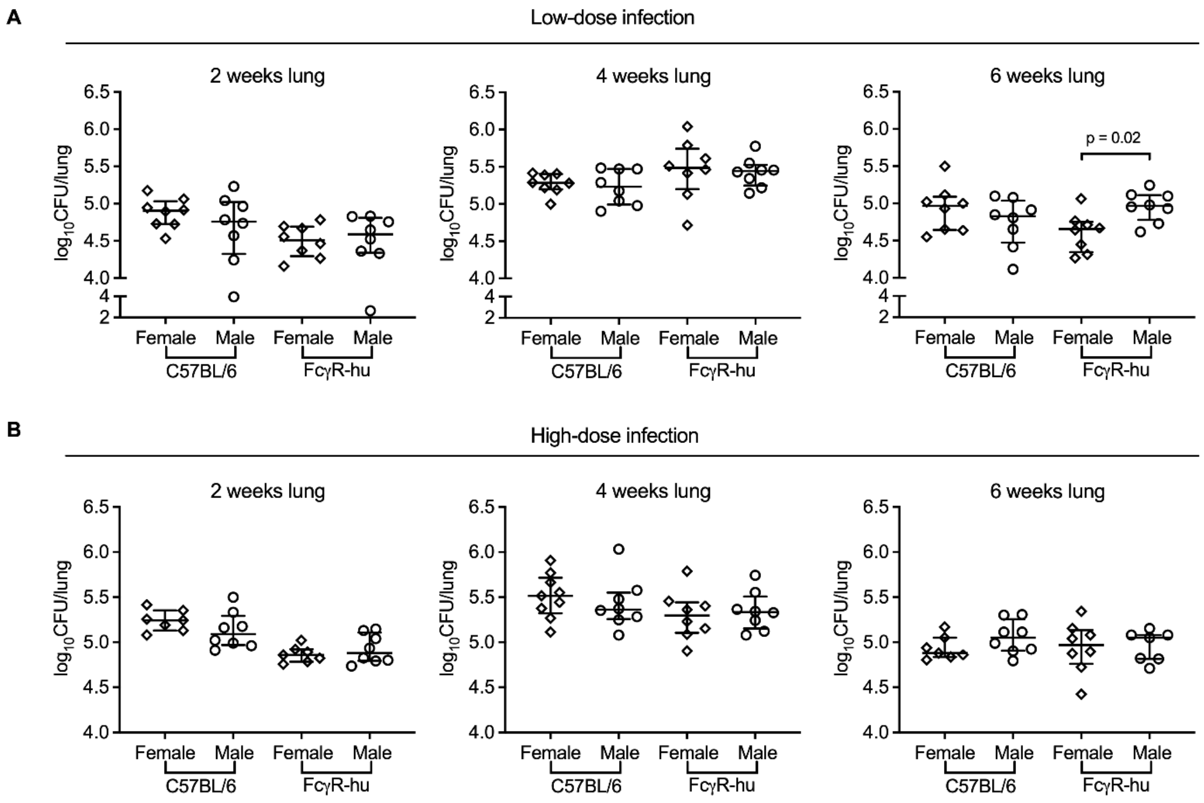

Supplemental Figure 3. Similar lung bacterial burden between female and male C57BL/6 and FcgR-hu mice post *Mtb* infection. Lung bacterial burden categorized by sex at two, four and six weeks after low-dose (78 CFU) **(A)** or high-dose (300 CFU) **(B)** *Mtb* (Erdman) infection. Lines and error bars represent medians with IQRs. Data set is same as shown in Fig. 6 but stratified by sex. Diamonds represent female mice; circles represent male mice. Mann-Whitney-U test.

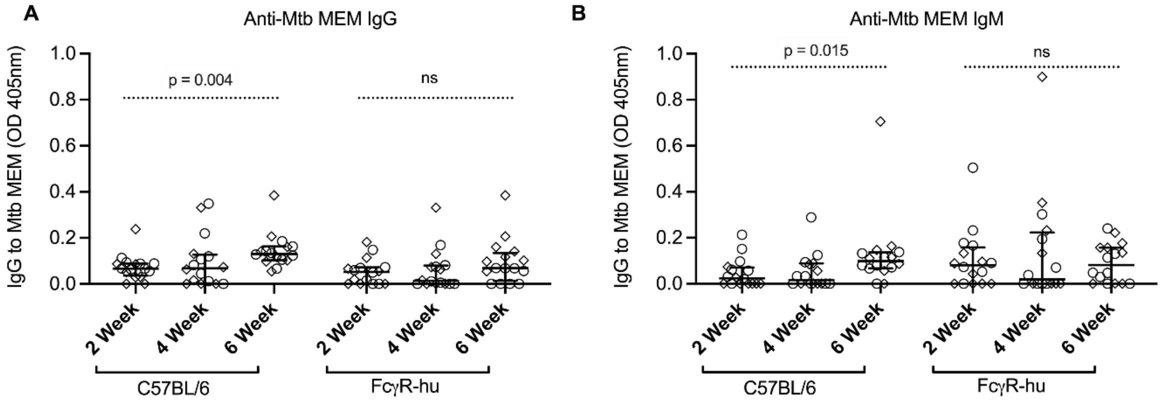

Supplemental Figure 4. Antibody responses to *Mtb* antigens are modest after *Mtb* infection in both C57BL/6 and FcγR-hu mice. IgG **(A)** and IgM **(B)** responses to *Mtb* membrane fractions (MEM) antibody in C57BL/6 and FcgR-hu mice at 2, 4, and 6 weeks post-*Mtb* infection (Erdman; mean lung CFU 300 ± 40 one day post-infection). OD405 values were subtracted by pre-infection values for each individual mouse. Lines and error bars represent medians with IQRs. Kruskal-Wallis test. Diamonds represent female mice; circles represent male mice. ns: not significant.

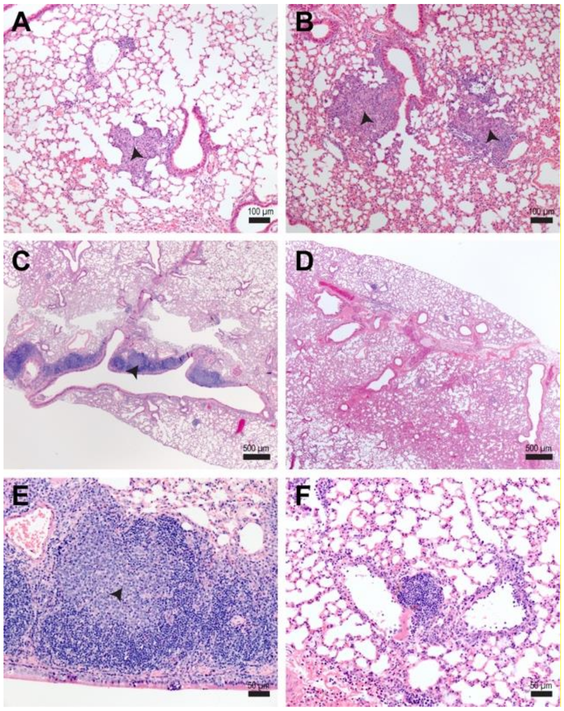

Supplemental Figure 5. Granulomatous reaction in the lungs of FcgR-hu and wild-type C57BL/6 mice two weeks after *Mtb* infection. Panels show sections of formalin-fixed, paraffin-embedded and H&E-stained lung tissues (5 µm) of mice two weeks post aerosolized infection with 300 CFU *Mtb* Erdman. Lung granulomatous reaction of FcgR-hu (A) and WT (B) mice, showing infiltration of immune cells consisting of macrophages, neutrophils, and lymphocytes (100x magnification). Aggregates are more robustly formed in the WT compared to FcgR-hu mice (denoted by dark arrow heads in B compared to A). The granulomatous reaction of the FcgR-hu mice also exhibits perivascular/peribronchiolar lymphocytic infiltrates containing cellular aggregates (devoid of macrophages or neutrophils) with features of germinal center (C; 25x magnification; E, 200x magnification); note the pale-staining region (dark arrow heads) of the cellular aggregates typical of proliferating B cells in a germinal center. Such germinal center-like aggregates were absent in the WT animals (D, 25x magnification; F, 200x magnification). Tissues of 5 mice (males and females) were examined for each group with 3 sections examined per mouse.

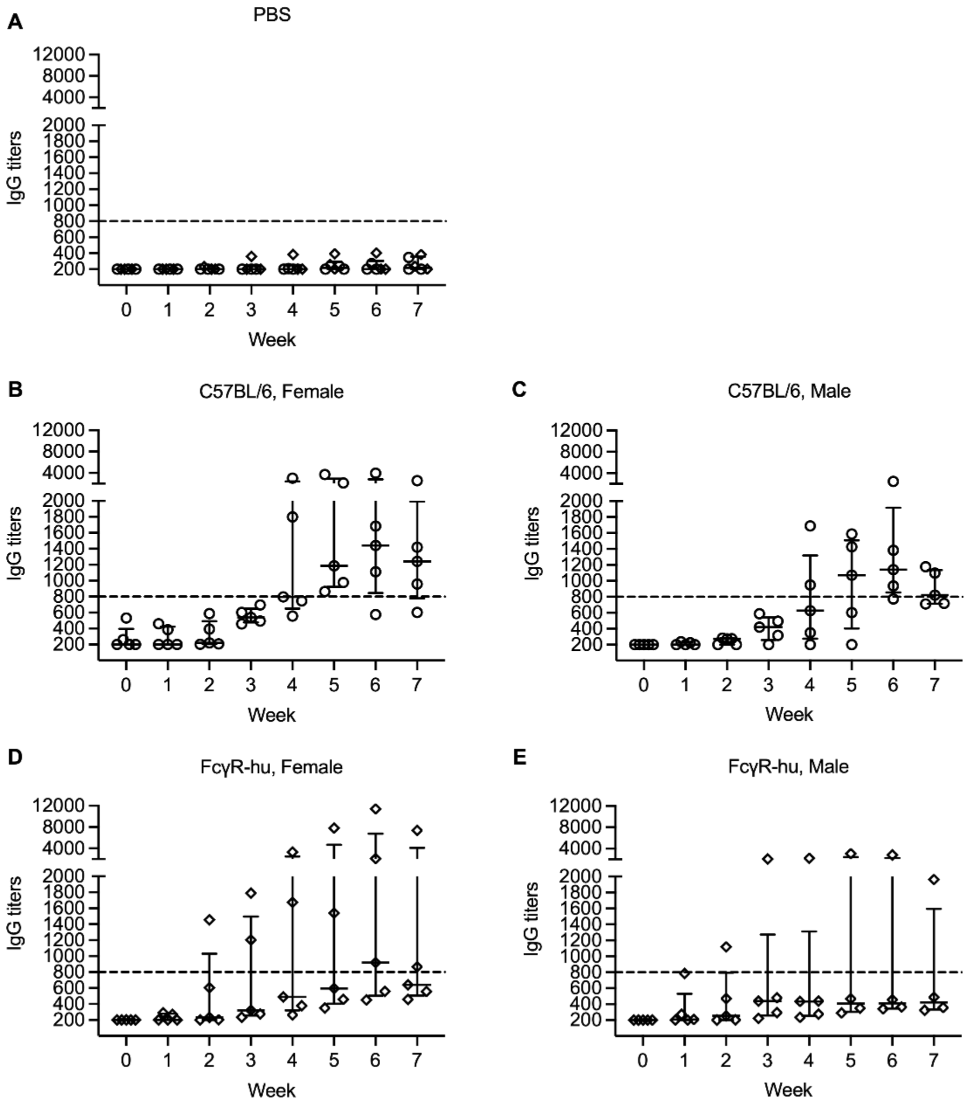

Supplemental Figure 6. Development of murine anti-human IgG after 4-5 weeks of passive human IgG transfer. Panels show anti-human IgG titers for **(A)** control group of female and male *Mtb*-infected C57BL/6 (n=3) and FcgR-hu mice (n=3) injected with PBS weekly, **(B)** female C57BL/6 (n=5), **(C)** male C57BL/6 (n=5), **(D)** female FcgR-hu (n=5), and **(E)** male FcgR-hu (n=5) *Mtb*-infected mice (150 CFU, Erdman) after weekly i.p. injections of 200 µg human IgG. Sera for week 0 was obtained before *Mtb* infection and first dose of human IgG or PBS. The dotted line shows two-fold titer increase from baseline, considered as the cutoff for seroconversion. Lines and error bars represent medians with IQRs.

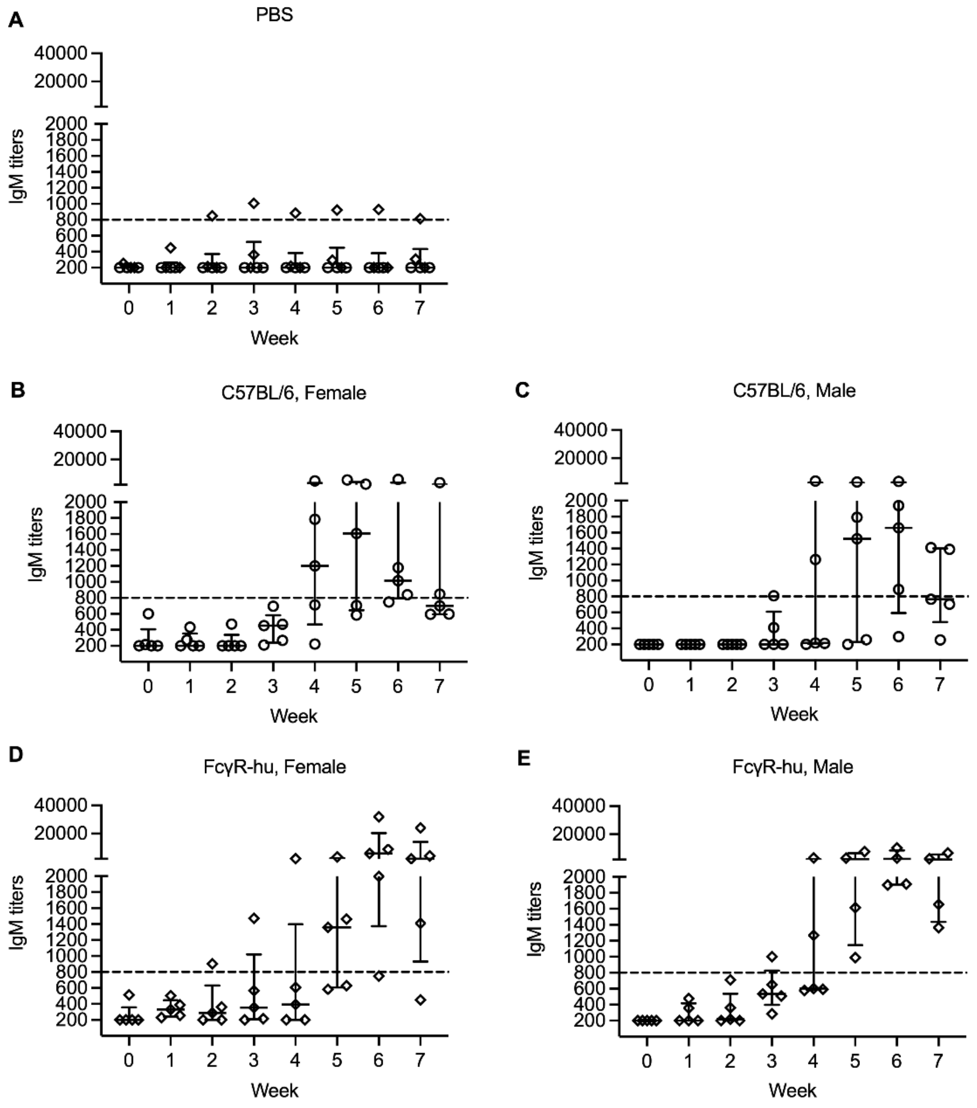

Supplemental Figure 7. Development of murine anti-human IgM after 4-5 weeks of passive human IgG transfer. Panels show anti-human IgM titers for **(A)** control group of female and male *Mtb*-infected C57BL/6 (n=3) and FcgR-hu mice (n=3) injected with PBS weekly, **(B)** female C57BL/6 (n=5), **(C)** male C57BL/6 (n=5), **(D)** female FcgR-hu (n=5), and **(E)** male FcgR-hu (n=5) *Mtb*-infected mice (150 CFU, Erdman) after weekly i.p. injections of 200 µg human IgG. Sera for week 0 was obtained before *Mtb* infection and first dose of human IgG at pre-injection (week 0) and weekly starting week 1. The dotted line shows a two-fold titer increase from baseline, considered as the cutoff for seroconversion. Lines and error bars represent medians with IQRs.
